# Generation of the induced pluripotent stem cell line ISMMSi061-A from a patient with ataxia, intention tremor, and hypotonia syndrome, childhood-onset

**DOI:** 10.1016/j.scr.2026.103938

**Published:** 2026-02-16

**Authors:** Norman N. Liu, Ruiqi Hu, Samuel J. Hubbard, Sophia E. Salemi, Erdene Baljinnyam, Priya Pradhan, Genevieve Smith, Bryn D. Webb, Samuele G. Marro

**Affiliations:** a Division of Genetics and Metabolism - Department of Pediatrics, Center of Precision Medicine, University of Wisconsin – School of Medicine and Public Health, USA; b Institute for Regenerative Medicine, Icahn School of Medicine at Mount Sinai, New York, NY, USA; c Nash Family Department of Neuroscience, Friedman Brain Institute, Icahn School of Medicine at Mount Sinai, New York, NY, USA; d Department of Genetics and Genomic Sciences, Icahn School of Medicine at Mount Sinai, New York, NY, USA

**Keywords:** POU4F1, Class IV POU domain-containing transcription factor, Ataxia intention tremor and hypotonia syndrome childhood onset, ATITHS, BRN3A

## Abstract

We have described a novel neurodevelopmental disorder caused by de novo, heterozygous pathogenic variants in the Pou domain, class 4, transcription factor 1 (*POU4F1*), now termed ataxia, intention tremor, and hypotonia syndrome, childhood-onset (ATITHS). This study reports the generation and characterization of an induced pluripotent stem cell (iPSC) line derived from peripheral blood mononuclear cells of a patient with ATITHS. The resulting iPSC line, ISMMSi061-A, harbors the *POU4F1* heterozygous variant c.271_281del;p.(Thr91Hisfs*254). The iPSCs display normal cell morphology, expression of pluripotency markers, normal karyotype, and the ability to differentiate into all three germ layers.

## 1. Resource utility

Pathogenic de novo heterozygous variants in *POU4F1* cause Ataxia, intention tremor, and hypotonia syndrome, childhood-onset (ATITHS), a disorder characterized by ataxia, intention tremor, and hypotonia. The iPSC line ISMMSi061-A derived from a patient carrying a heterozygous pathogenic variant in *POU4F1* provides a valuable cellular model for exploring the function and mechanism of POU4F1 and further investigating ATITHS (see Table 1).

## 2. Resource details

Hereditary ataxias are a highly heterogeneous group of disorders with symptoms manifesting as slowly progressive incoordination of movement and gait (Jayadev and Bird, 2013). Although more than 150 genes have been identified as causative of hereditary ataxias, including genes with autosomal recessive, autosomal dominant, X-linked, and mitochondrial inheritance, many causative disease genes have yet to be recognized and many affected patients remain without an underlying molecular diagnosis (Parodi, 2018). We identified a novel hereditary ataxia syndrome named Ataxia, intention tremor, and hypotonia syndrome, childhood-onset (ATITHS, MIM #619352), caused by de novo heterozygous variants in the *POU4F1* gene and characterized by ataxia, intention tremor, and hypotonia (Webb, 2021).

POU4F1, also known as BRN3A, is a class IV POU domain-containing transcription factor expressed in the developing nervous system. The POU domain is a bipartite structure composed of two subdomains, a POU-specific domain and a POU-homeodomain, separated by a non-conserved linker region of 15–55 amino acids (Herr, 1988; Malik et al., 2018). Members of the class IV POU domain family include *C. elegans unc-86*, which is expressed exclusively in neuroblasts and neurons, as well as the human *POU4F1*, *POU4F2*, and *POU4F3* genes, which show region-specific expression in the brainstem, retina, dorsal root ganglia, and trigeminal ganglia (Xiang, 1996). In the mouse, *Pou4f1* is expressed in discrete neuronal populations, including the medial habenula, red nucleus, inferior olivary nucleus, and superior colliculus (Fedtsova and Turner, 1995; Gerrero, 1993). Targeted disruption of *Pou4f1* results in a severe phenotype characterized by decreased suckling, absence of rhythmic jaw opening, uncoordinated limb and truncal movements, impaired righting, and death within 24 h of birth, whereas *Pou4f1*^+/−^ mice are indistinguishable from wild-type littermates (Xiang, 1996). Consistent with these findings, *Pou4f1*^−/−^ mice exhibit selective loss of neurons in the trigeminal ganglia, medial habenula, red nucleus, inferior olivary nucleus, and nucleus ambiguous (Xiang, 1996; McEvilly, 1996; Serrano-Saiz, 2018).

In this study, we generated human induced pluripotent stem cells (iPSCs) from peripheral blood mononucleate cells (PBMCs) obtained from an index patient with ATITHS carrying the de novo heterozygous pathogenic variant c.271_281del;p.(Thr91Hisfs*254) in *POU4F1* (NM_006237.3) (Webb, 2021). PBMCs were expanded and cryopreserved (M1619E), and were transduced with four factors (KLF4, OCT4, SOX2, and c-MYC) using non-integrating Sendai virus and plated on mitotically inactivated mouse embryonic fibroblasts (MEFs) feeder. Multiple single clones were manually picked, expanded on MEF and gradually weaned to feeder-free conditions. Three iPSC clones were subsequently expanded, and one clone (CSI2444A) was fully characterized and registered as *ISMMSi061-A.*

**Fig. 1.**
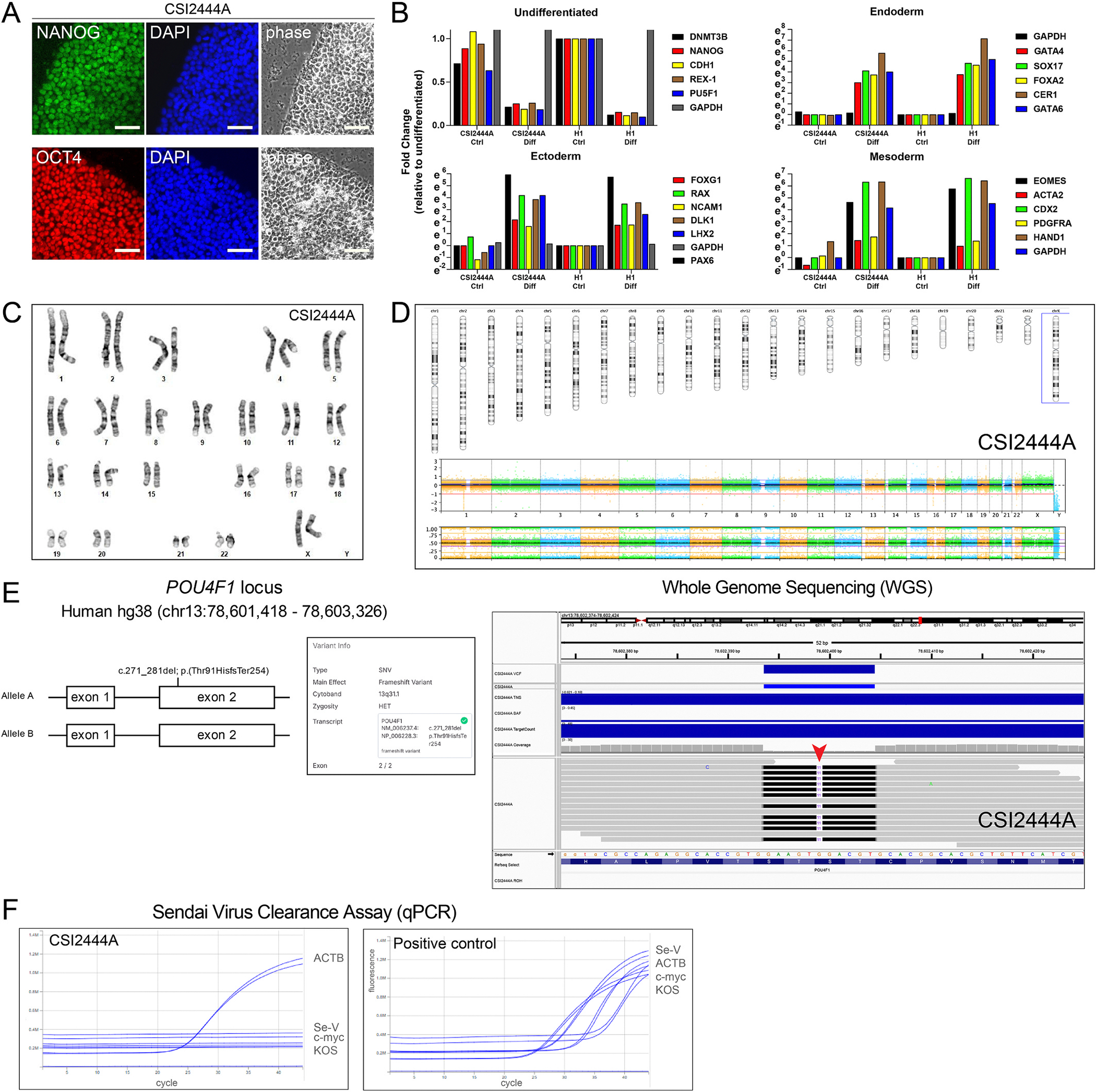
Characterization and validation of the patient-derived iPSC line ISMMSi061-A harboring the heterozygous POU4F1 c.271_281del variant.

*ISMMSi061-A* exhibits the classical iPSC morphology and expresses common pluripotency markers (Fig. 1A). *ISMMSi061-A* iPSCs differentiate into all three germ layers (Fig. 1B), tested negative for mycoplasma contamination, and have normal G-banded karyotype (Fig. 1C). No CNV or LOH > 1.5 Mb was detected on somatic chromosomes using SNP-array CNV analysis (Fig. 1D). Using whole genome sequencing, we confirmed the presence of the heterozygous variant in *POU4F1* (c.271_281del;p.(Thr91Hisfs*254)) in genomic DNA extracted from ISMMSi061-A iPSCs (Fig. 1E). Finally, Sendai virus clearance was confirmed by RT-qPCR (Fig. 1F).

Overall, this patient iPSC line can be used to study the effects of *POU4F1* mutations in human cell models. These cells are banked for distribution at the Stem Cell Engineering Core of the Icahn School of Medicine at Mount Sinai (New York, NY).

## 3. Materials and methods

### 3.1. Case details and study approval

The recruited patient (proband 4) has been identified to have the de novo heterozygous pathogenic variant c.271_281del;p. (Thr91Hisfs*254)in *POU4F1,* and a diagnosis of ATITHS (Webb, 2021). For cell line generation, peripheral blood was obtained from the patient, and a PBMCs were isolated and cryopreserved (M1619E). The study protocol was approved by the Icahn School of Medicine at Mount Sinai and the University of Wisconsin School of Medicine and Public Health. Written informed consent was obtained from the subject.

### 3.2. Cell culture and iPSC generation

PBMCs (M1619E) were expanded in Erythroid Expansion medium (EEM) composed of StemSpan SFEM II (STEMCELL Technologies, #09605) and StemSpan Erythroid Expansion Supplement (STEMCELL Technologies, #02692). On day 0, 150,000 cells were transduced with KLF4, OCT4, SOX2, C-MYC Sendai virus (CytoTune-iPS 2.0 Reprogramming Kit, Thermo Fisher, #A16517) with a MOI of 2.5. Twenty-four hours post-transduction, cells were washed and medium was replaced with fresh EEM. On day 3, cells were replated into plates containing mitotically inactivated mouse embryonic fibroblasts (MEFs) and 0.2% gelatin solution (Sigma, #G2500) with embryonic stem (ES) medium composed of knockout-DMEM-F12 (Thermo Fisher, #12660012), 20% knock-out serum replacement (Thermo Fisher, #10828028), 1X GlutaMAX Supplement (Thermo Fisher, #35050061), 1X MEM non-essential amino acids (Thermo Fisher, #11140050), 115 μM 2-mercaptoethanol (Sigma, #M3148), and 20 ng/ml of Recombinant Human FGF basic (Biotechne, #233-FB). Medium was exchange daily to ES medium with 500 μM valproic acid (Sigma, #P4543) until the first colonies emerged (day 12–20). On days 16–18, single clones were manually picked and plated on MEF feeder. Multiple iPSC single clones were manually picked, expanded on MEFs and ES media and gradually weaned to feeder-free conditions. The iPSC cultures were maintained on Geltrex (Thermo Fisher, #A1413302), kept in mTeSR Plus (STEMCELL Technologies, #100-0276) and passaged using enzyme-free DPBS − 0.5 mM EDTA (Thermo Fisher, #4190250) at a 1:6 to 1:20 split ratio. All cells were cultured at 37°C in humidified atmosphere containing 5% CO2.

### 3.3. Immunocytochemistry

Cells were fixed with 4% paraformaldehyde at room temperature for 10 min, permeabilized with 0.2% Triton-X in PBS for 5 min. Cells were then incubated with blocking buffer (PBS with 5% calf serum) for 1hr, followed by primary antibody incubation overnight at 4°C, and secondary antibody incubation for 1hr at room temperature. Antibody information can be found in Table 2.

### 3.4. Trilineage differentiation assay

Trilineage differentiation was performed using the STEMdiff Trilineage Differentiation Kit (STEMCELL Technologies, #05230) following manufacturer’s recommendations. RNA was isolated from the differentiated and undifferentiated cells at day 5 (endoderm and mesoderm) and day 7 (ectoderm). Markers expression was assessed via RNA sequencing (Illumina HiSeq, 2×150bp with PolyA selection), analyzed with Kallisto and DESeq2 software (Love et al., 2014; Bray, 2016).

### 3.5. Karyotyping

Chromosomal G-band analysis was performed at WiCell Research Institute, according to the International System for Human Cytogenetic Nomenclature. iPSCs were tested at passage 18, with 20 cells in metaphase counted for the analysis. Band resolution was 400–500 bands. CNV analysis analysis was performed by the Stem Cell Engineering Core at Mount Sinai using Infinium Global Diversity Array-8 v1.0 (Illumina). Data analysis was conducted with VIA software (Bionano). CNVs of LOH encompassing regions smaller than 1.5 Mb were not considered.

### 3.6. Short tandem repeat (STR)

STR analysis was carried out by the WiCell Characterization Lab (Madison, WI) using the PowerPlex 16 HS system (Promega) to analyze STR polymorphisms for 15 loci plus amelogenin.

### 3.7. Whole genome sequencing (WGS)

Whole genome sequencing (WGS) was performed by Azenta Life Sciences (South Plainfield, NJ, USA). Genomic DNA was sequenced as paired-end 150 bp reads on an Illumina platform to a mean genome-wide coverage of approximately 30x. Reads were aligned to the human reference genome (GRCh38/hg38), and single-nucleotide variants and small insertions/deletions were identified using the Illumina DRAGEN pipeline. Variant annotation and pathogenicity assessment were performed using eMedgene (Franklin, MA, USA). Variants were evaluated according to American College of Medical Genetics and Genomics and Association for Molecular Pathology (ACMG/AMP) guidelines, incorporating population allele frequency, predicted functional impact, and relevance to the disease phenotype.

### 3.8. Mycoplasma test

Mycoplasma contamination analysis was carried out via MycoAlert PLUS Mycoplasma Detection Kit (Lonza, LT07–710) according to the manufacturer’s protocol.

### 3.9. Software

Fig. 1 was created with Adobe Illustrator. Graphs with GraphPad Prism, phase and fluorescence images with ImageJ.

## Supplementary Material

1

2

## Figures and Tables

**Table 1:** Characterization and validation.

Classification	Test	Result	Data

Morphology	Photography	Typical primed pluripotent human stem cell morphology	Fig. 1A
Phenotype	Qualitative analysis: Immunocytochemistry	Expression of pluripotency markers: OCT4 and NANOG markers	Fig. 1A
	Quantitative analysis: mRNA expression	Expression of pluripotency markers: NANOG, REX1, OCT4, DNMT3B, CDH1	Fig. 1B
*mtDNA analysis* (IF APPLICABLE)	*N/A*	*N/A*	*N/A*
Identity	Whole Genome Sequencing	Patient’s pathogenic variant confirmed	Fig. 1E
	STR analysis	15 loci plus amelogenin	Supplementary file 1, submitted in the archive with journal
Mutation analysis	Sequencing	Heterozygous variant identified	Fig. 1E
	Southern Blot OR WGS	N/A	N/A
Microbiology and virology	Mycoplasma	Negative	Supplementary file 3
Differentiation potential	Direct differentiation	Upregulation of trilineage markers	Fig. 1B
Donor screening (OPTIONAL)	HIV 1 + 2 Hepatitis B, Hepatitis C	–	not shown but available with author
Genotype additional info	Blood group genotyping	–	not shown but available with author
(OPTIONAL)	HLA tissue typing	–	not shown but available with author

**Table 2:** Reagents details.

	Antibodies used for immunocytochemistry/flow-cytometry			
	Antibody	Dilution	Company Cat #	RRID

Pluripotency marker	Goat anti-NANOG	1:200	R&D Systems Cat# AF1997	RRID: AB_355097
Pluripotency marker	Mouse anti OCT4	1:250	Santa Cruz Biotechnology Cat# sc-5279	RRID: AB_10559812
Secondary antibody	AlexaFluor-555 Donkey anti-mouse	1:500	Jackson ImmunoResearch Labs Cat# 715-565-150	RRID: AB_3095479
Secondary antibody	AlexaFluor-488 Donkey anti-goat	1:500	Jackson ImmunoResearch Labs Cat# 705-545-003	RRID: AB_2340428

**Resource Table:** 

Unique stem cell line identifier	ISMMSi061-A
Alternative name(s) of stem cell line	CSI2444A
Institution	Icahn School of Medicine at Mount Sinai
Contact information of distributor	Bryn D. Webb; bdwebb@wisc.edu; Samuele G. Marro; samuele.marro@mssm.edu
Type of cell line	Induced pluripotent stem cells
Origin	Human
Additional origin info required for human ESC or iPSC	Age: 25 years Sex: Female Ethnicity if known: Caucasian
Cell Source	PBMCs (M1619E)
Clonality	Clonal
Method of reprogramming	PBMCs reprogrammed with four human factors (KLF4, OCT4, SOX2, and c-MYC) using non-integrating Sendai virus (CytoTune-iPS 2.0 Sendai Reprogramming Kit, Invitrogen)
Genetic Modification	YES
Type of Genetic Modification	Heterozygous 11-bp deletion (c.271_281del) in *POU4F1* (RefSeq NM_006237.3), resulting in a frameshift with predicted premature termination p.(Thr91Hisfs*254)
Evidence of the reprogramming transgene loss (including genomic copy if applicable)	q-PCR Sendai Clearance Assay
Associated disease	Ataxia, intention tremor, and hypotonia syndrome, childhood-onset (ATITHS)
Gene/locus	POU Domain, Class 4, Transcription Factor 1 (POU4F1) GRCh38/hg38; chr13:78,598,362–78,603,552
Date archived/stock date	5/22/2025
Cell line repository/bank	Stem Cell Engineering Core (RRID: SCR_027503) at the Icahn School of Medicine at Mount Sinai
Ethical approval	Holder of the original Donor Information Sheet: Bryn D. Webb, MD; bdwebb@wisc.edu; University of Wisconsin-Madison

## Data Availability

Data will be made available on request.

## Appendix A. Supplementary data

Supplementary data to this article can be found online at https://doi.org/10.1016/j.scr.2026.103938.
