# Supplementary material for: Generation of the induced pluripotent stem cell line ISMMSi061-A from a patient with ataxia, intention tremor, and hypotonia syndrome, childhood-onset
Source: Stem Cell Res. Author manuscript; Available in PMC 2026 Apr 18. (PMC13091698; doi:10.1016/j.scr.2026.103938)
Supplement: 1 [file NIHMS2158095-supplement-1.pdf]

Chromosome Analysis Report: 110081

Date Reported: December 13, 2025

Cell Line: CSI2444A

Submitted Passage #: 18

Date of Sample: 12/8/2025

Specimen: Human IPSC

Results: 46,XX

Cell Line Sex: Female

Reason for Testing: None Given

Investigator: Bryn Webb, UW Madison

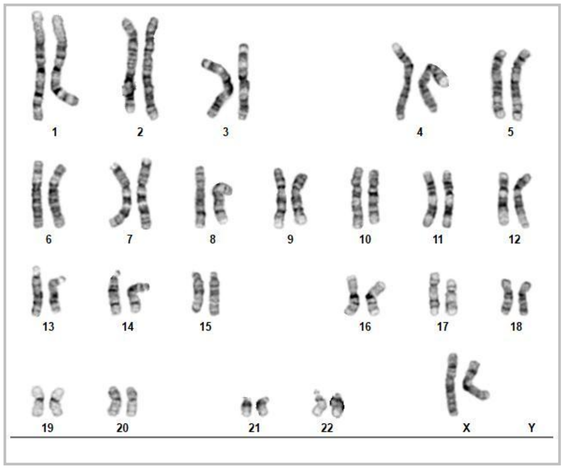

Cell: 61

Slide: G02

Slide Type: Karyotype

Total Counted: 20

Total Analyzed: 8

Total Karyogrammed: 4

Band Resolution: 400 - 500

Interpretation:

This is a normal karyotype; no clonal abnormalities were detected at the stated band level of resolution.

Completed by: Erica Schutter, CG(ASCP)

Reviewed and Interpreted by: Justin Schleede, PhD, FACMG

For internal use only

Date:________________________ Sent By:____ Sent To:_____________________ QC Review By: ____

Limitations: This assay allows for microscopic visualization of numerical and structural chromosome abnormalities. The size of structural abnormality that can be detected is >3-10Mb, dependent upon the G-band resolution obtained from this specimen. For the purposes of this report, band level is defined as the number of G-bands per haploid genome. It is documented here as "band level", i.e., the range of bands determined from the four karyograms in this assay. Detection of heterogeneity of clonal cell populations in this specimen (i.e.,mosaicism) is limited by the number of metaphase cells examined, documented here as "# of cells counted".

This assay was conducted solely for listed investigator/institution. The results of this assay are for research use only. Unless otherwise mutually agreed in writing, the services provided to you hereunder by WiCell Research Institute, Inc. ("WiCell") are governed solely by WiCell's Terms and Conditions of Service, found at www.wicell.org/privacyandterms. Any terms you may attach to a purchase order or other document that are inconsistent, add to, or conflict with WiCell's Terms and Conditions of Service are null and void and of no legal force or effect.
