# Supplementary material for: Generation of the induced pluripotent stem cell line ISMMSi061-A from a patient with ataxia, intention tremor, and hypotonia syndrome, childhood-onset
Source: Stem Cell Res. Author manuscript; Available in PMC 2026 Apr 18. (PMC13091698; doi:10.1016/j.scr.2026.103938)
Supplement: 2 [file NIHMS2158095-supplement-2.pdf]

Mycoplasma Assay Report

Assay Information

The Stem Cell Engineering Core Myco Assay detects all common mollicute contaminations (Mycoplasma, Acholeplasma, Entomoplasma and Spiroplasma; except Ureaplasma) in the supernatant of eukaryotic Cells. The enzymatic assay is based on the activity of mycoplasma enzymes which are found in all six of the main mycoplasma cell culture contaminants and the vast majority of 180 mycoplasma species, but are not present in eukaryotic cells. Viable mycoplasma in a test sample (cell supernatant) are lysed and the enzymes react with the substrate, catalyzing the conversion of ADP to ATP. The ATP is then transferred into a light signal via the luciferase enzyme. By measuring the level of ATP in a sample both before (read A) and after the addition of the substrate (read B), a ratio can be obtained which is indicative of the presence or absence of mycoplasma.

Samples with **ratio < 0.9** are considered negative.

User/PI: **Webb/Marro**

Assay Date: **September 21, 2025**

Assay Results:

Samples	Sample ID	A reagent	B substrate	Ratio	Result
no 1	CSI2444A	3012	1283	0.43	NEGATIVE
Negative Ctrl		3549	1392	0.39	
Positive Ctrl		4324	187302	43.32	
